# Prediction of remission after metabolic surgery using a novel scoring system in type 2 diabetes – a retrospective cohort study

**DOI:** 10.1186/s40200-014-0089-y

**Published:** 2014-08-22

**Authors:** Surendra Ugale, Neeraj Gupta, Kirtikumar D Modi, Sunil K Kota, Vasisht Satwalekar, Vishwas Naik, Modukuri Swapna, KVS Hari Kumar

**Affiliations:** Department of Advanced Laparoscopy and Metabolic Surgery, Kirloskar Hospital, Hyderabad, Andhra Pradesh India; Department of Endocrinology, Medwin Hospital, Hyderabad, AP India; Department of Endocrinology, Endocare Hospital, Vijayawada, AP India; Department of Endocrinology, Command Hospital, Chandimandir, 134107 Haryana India

**Keywords:** Type 2 Diabetes, Ileal interposition, Sleeve gastrectomy, Diabetes remission, Metabolic surgery

## Abstract

**Background:**

Remission of diabetes is seen in more than 60% of patients after bariatric surgery. There is extensive variability in the remission rates between different surgical procedures. We analyzed our database and aimed to develop an easy scoring system to predict the probability of diabetes remission after two surgical procedures i.e. Ileal Interposition coupled with Sleeve Gastrectomy (IISG) or Diverted Sleeve Gastrectomy (IIDSG).

**Methods:**

In this retrospective study, we analyzed records pertaining to patients who underwent IISG (n = 46) and IIDSG (n = 29). The primary outcome measure was diabetes remission (A1c <6.5% and not requiring hypoglycemic drugs). We identified seven preoperative clinical variables (age, duration of diabetes, body mass index, micro and macrovascular complications, use of insulin and stimulated C-peptide) based on our previous reports to be included in the diabetes remission score (DRS). The DRS score (7 – 14) was compared between the patients with and without remission in both the surgery groups.

**Results:**

Mean DRS in patients who underwent IISG was 9.2 ± 1.4. Twenty one (46%) had a remission in diabetes. DRS was significantly lower in patients with remission than patients without remission (8.1 ± 0.8 versus 10.2 ± 0.9, p < 0.0001). Mean DRS in patients who underwent IIDSG was 10.4 ± 1.3. Twenty one (72%) had a remission in diabetes. DRS was significantly lower in patients with remission than patients without remission (9.7 ± 0.8 versus 12.0 ± 0.5, p < 0.0001). Patients with a DRS ≥ 10 in IISG group and more than 12 in IIDSG group did not get into remission.

**Conclusion:**

Preoperative DRS can be a useful tool to select the type of surgical procedure and to predict the postoperative diabetes remission.

**Trial registration:**

NCT00834626.

## Background

Bariatric surgery in patients with type 2 diabetes is known to produce remission in a majority of cases. The improvement is not limited to diabetes alone and includes hypertension, dyslipidemia and obstructive sleep apnea as well. The magnitude and onset of this improvement outweighs the weight loss leading to the concept of ‘metabolic surgery’ rather than bariatric surgery [1]. The observed metabolic benefits lead to the extension of this concept to non obese patients also [2-5]. Bariatric surgical procedures are primarily divided into restrictive and malabsorptive types. The most common surgical procedures are sleeve gastrectomy (SG), gastric banding, Roux-en-Y-Gastric Bypass (RYGB) and Jejuno-Ileal Bypass (JIB). The observed remission in diabetes after these surgeries is reported to range between 45-95% [6].

The standard procedures have been modified to achieve maximum remission with minimal long term complications. The modified surgical techniques are Ileal Interposition (II), Diverted Sleeve Gastrectomy (DSG), Sleeve Gastrectomy with Duodeno-jejunal Bypass (SG + DJB), Mini-Gastric Bypass (MGB) and Single Anastomosis Duodeno-Ileal (SADI) procedure [6]. The results are often incomparable between procedures due to the differences involved in patients and the spectrum of diabetes. The proposed predictive factors for remission and other outcomes includes age, diabetes duration, insulin reserve& requirement, body mass index (BMI), ethnicity and number of preoperative medications required to manage blood glucose [7-12]. We observed a significant variability in the remission rates using IISG and IIDSG as reported previously [13-17]. A good predictive model is essential in helping the patients and clinicians to decide about the probability of remission based on preoperative criteria. We developed a novel scoring system known as Diabetes Remission Score (DRS) based on the previous database. Our intention is to develop a simple, effective and useful method to help the surgical teams in predicting the diabetes remission rates postoperatively.

## Methods

The present study is a retrospective analysis of all the patients with poorly controlled type 2 diabetes mellitus (T2DM), who were subjected to the laparoscopic procedures of IISG or IIDSG at Kirloskar Hospital, Hyderabad, India. IISG was started in February 2008 and IIDSG was started in March 2010 and the study was duly approved by the hospital’s ethics committee (Institutional Review Board, Kirloskar Hospital, Hyderabad) and registered (NCT00834626). Informed consent was obtained from all patients participating in the study. Study participants were explained specifically about the benefits pertinent to nonobese subjects and the potential risks involved. The patient selection criteria and surgical techniques have been described in detail previously (13 – 17). Briefly, we included patients having T2DM of more than 1 year duration (>5 years duration for IIDSG), aged between 25 and 70 years, poor glycemic control with HbA1C > 7% on the optimum dosage of insulin ± oral hypoglycemic agents (OHA), stable weight, BMI ≥20 kg/m^2^ (>18.5 kg/m^2^ for IIDSG) and stimulated C-peptide level >1.5 ng/ml. The decision of mode of surgery (IISG or IIDSG) was taken by the surgical team in consultation with the endocrinologist and the patient’s preference. The study is a retrospective analysis of the patients profile precluding the possibility of random allocation into each group. We excluded patients with type 1 diabetes mellitus, undetectable fasting C-peptide, ketoacidosis in past 6 months, pregnancy and coexisting severe hepatic, pulmonary and psychiatric diseases.

### Surgical technique

#### IISG

**Figure 1:**
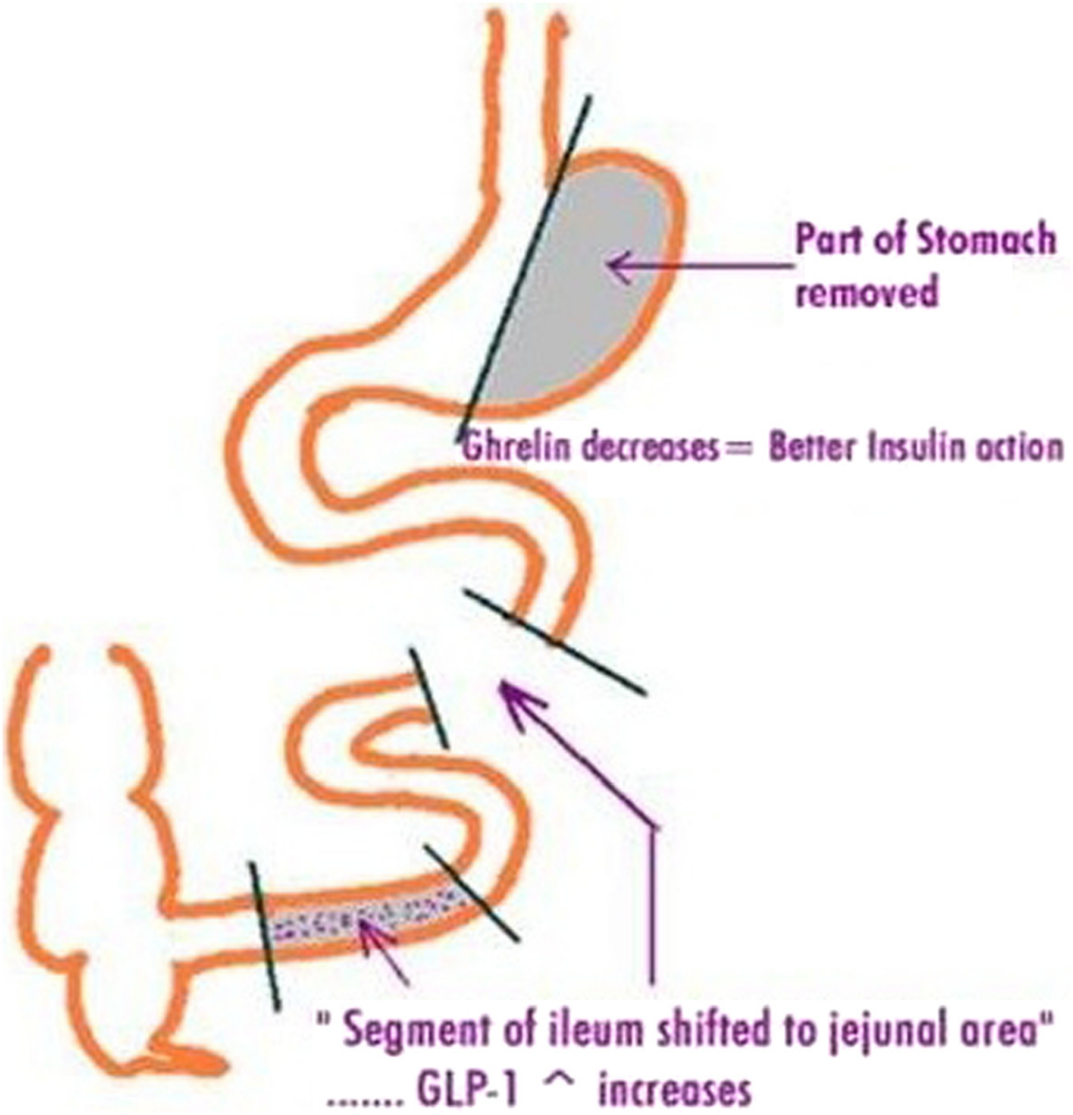
**Schematic diagram of Ileal Interposition with Sleeve Gastrectomy.**

The surgical procedure involved the creation of a 170-cm segment of ileum, starting at 30 cm proximal to the ileo-cecal junction. This segment was interposed into the jejunum, which was divided between 20 and 50 cm from the ligament of Treitz. All three anastomoses were performed side by side with an endo-GIA stapler (Ethicon Endo-surgery, Cincinnati, OH, USA). A variable sleeve gastrectomy was performed after devascularization of the greater curvature from the antrum to the fundus area as shown in Figure 1. The lumen of the stomach was adjusted by a 32–60 French calibrator (Romsons International, New Delhi, India) that was placed along the lesser curvature. The size of the bougie is determined for each patient individually depending on the baseline body weight, BMI and the anticipated amount of weight loss. The size between 32 F to 38 F was used in majority of the patients. The endo-GIA stapler with 60-mm cartridges were used for resection. Nonobese patients were subjected to only fundectomy, leaving a good volume of residual stomach for normal food intake. The fundectomy was carried by removing the fundus and with the proximal limit of dissection 3 cm to cardia.

#### IIDSG

**Figure 2:**
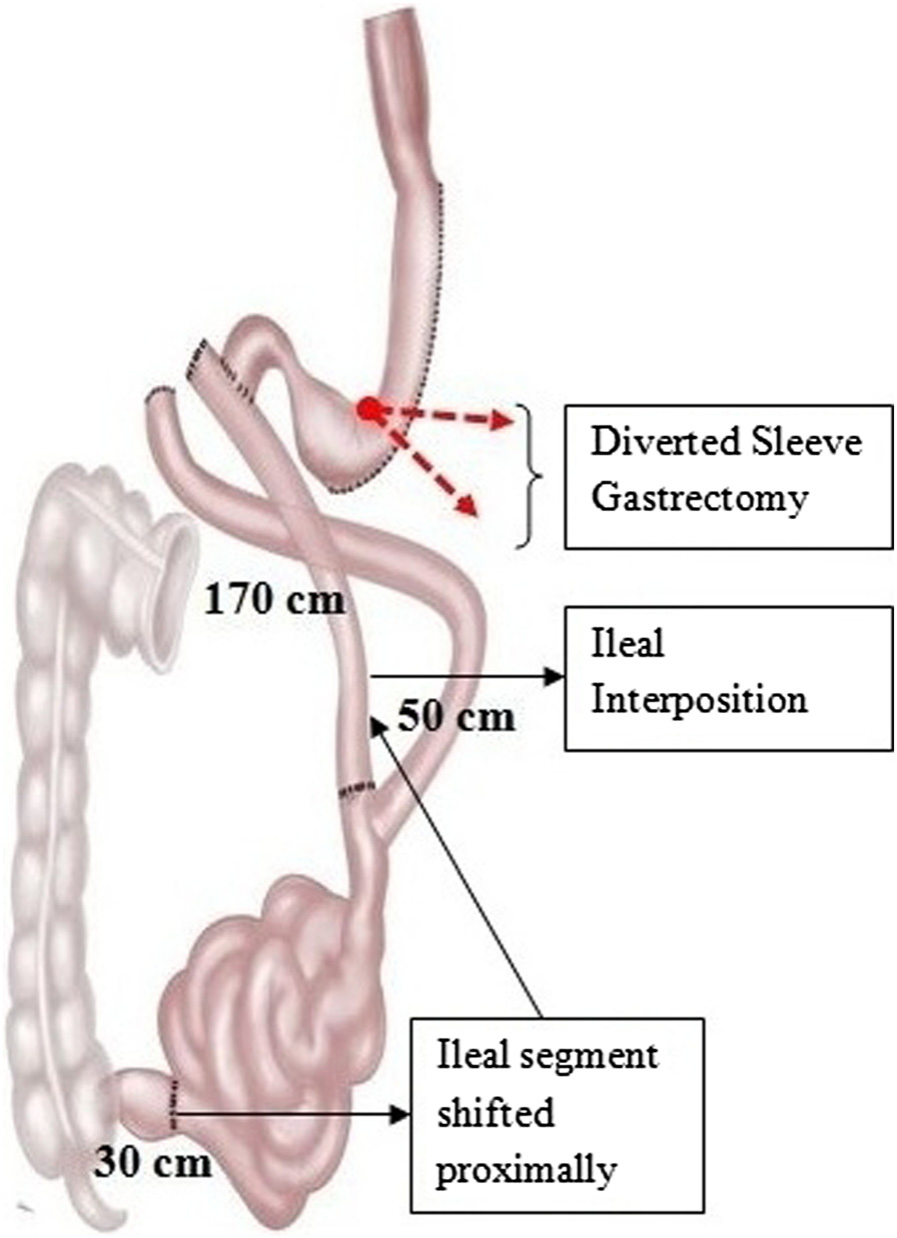
**Schematic diagram of Ileal Interposition with Diverted Sleeve Gastrectomy.**

A variable sleeve gastrectomy was performed after devascularization of the greater curvature from the antrum to the fundus area as described above. The duodenum was transected using a 60-mm linear stapler with a blue cartridge. The role of surgical stapler is to provide tissue approximation and hemostasis without leading to necrosis and destruction. The tissue thickness varies in the stomach and it is important to select the most appropriate staple cartridge to accommodate the underlying tissue [18]. The staple cartridges are color coded as gray, white, blue, gold, green and black in the ascending order of closed staple height dimensions. The gastric pouch and proximal duodenum were then transposed through a window created at the root of the transverse mesocolon. A distal ileal segment of 170 cm was transected along with its mesentery pedicle, measured 30 cm proximal to the ileocecal valve. Continuity of small bowel is restored by ileo-ileal anastomosis and the proximal end of this transected ileal segment was anastomosed to the proximal duodenum as shown in Figure 2. A point in the jejunum 50 cm from the ligament of Treitz was measured and anastomosed to the distal part of the interposed ileum, side to side, using stapler.

Post operatively each participant was followed up at 1, 3, 6 ,9 and 12 months in the 1st year and every 6 months thereafter. They were subjected to clinical and biochemical assessment. HbA1C <6.5% (without requiring any medication for diabetes control) was defined as the criterion for remission in diabetes. Our study protocol was started in 2008 and the ADA defined diabetes remission into partial and complete in 2009 [19]. Hence, our definition is similar to that of partial remission given by the ADA.

### Statistical analysis & scoring system

All outcome measures were evaluated prospectively from the third month onward at every visit. The seven variables to be included in the DRS have been derived from our previous reports. The details of the DRS components are given in Table 1. DRS was calculated in all the patients retrospectively and compared between IISG and IIDSG procedures. We used Graphpad Quickcalcs software (Graphpad Software Inc, La Jolla, CA, USA) for statistical calculations. The continuous variables were described in the form of mean ± standard deviation and comparison between the groups was done using unpaired student’s t test. All tests were two sided and a p value less than 0.05 were considered significant.

**Table 1:** **Diabetes Remission Score Calculator**

**Parameter**	**Score**	
	**1**	**2**
**Age (yr)**	30 – 60	< 30 or >60
**Body Mass Index (kg/m** ^**2**^ **)**	≥ 27	< 27
**Duration of T2DM (yr)**	< 10	≥ 10
**Microvascular complications**	No	Yes
**Macrovascular complications**	No	Yes
**Preoperative Insulin use**	No	Yes
**Stimulated C-peptide (ng/mL)**	≥ 4	< 4

## Results

Forty six patients underwent IISG and 29 patients underwent IIDSG surgical procedures. The baseline demographic parameters are shown in Table 2 and the operation details in Table 3. Mean DRS in patients who underwent IISG (n = 46) was 9.2 ± 1.4 (Range: 7–13). Twenty one (46%) had a remission in diabetes. In the same group, patients with BMI ≥ 35 kg/m2, had a remission rate of 85%. Their mean DRS was 8.9 ± 1.7 versus mean DRS of 9.5 ± 1.1 in patients with BMI less than 35 (p = 0.07). DRS was significantly lower in patients with remission than patients without remission (8.1 ± 0.8 versus 10.2 ± 0.9, p < 0.0001). All the patients with DRS 7–8 achieved remission of diabetes, while only 35% patients with DRS 9 had diabetes remission. There was no remission in diabetes in patients with baseline DRS ≥ 10 in IISG group as shown in Table 4.

**Table 2:** **Comparison between 2 groups regarding the clinical profile and complications**

**Parameter**	**Units**	**IISG group n = 46**	**IIDSG group n = 29**
Age	years	51.7 (13.3)	57.6 (11.5)
Sex	M:F	29 : 17	20 : 9
BMI	Kg/m^2^	23.4 (4.5)	25.6 (4.5)
DM duration	Years	9.9 (4.8)	10.1 (5)
Duration of insulin use	Years	3.3 (2.9)	3.6 (2.8)
Daily Insulin dose	Units	28.2 (10.6)	75.5 (12)
Daily Insulin dose	U/Kg	0.45 (0.15)	1.1 (0.1)
Retinopathy	Number	21	13
Acanthosis	Grade	0.83 (0.9)	1.1 (0.8)
HbA1c	%	8.1 (0.59)	9 (0.78)
HDL cholesterol	mg/dL	40.2 (5.7)	41 (5)
LDL cholesterol	mg/dL	92.7 (18.5)	96 (18.9)
Triglycerides	mg/dL	106.7 (37.2)	107 (44)
Serum Iron	μg/dL	104.5 (22.4)	112.1 (18.7)
Serum Ferritin	ng/mL	44.7 (18.5)	53.3 (12.2)
Vitamin B_12_	pg/mL	423.3 (102.4)	397.9 (92.9)

Mean (S.D).

**Table 3:** **Comparison between 2 groups regarding the surgical procedure and complications**

**Parameter**	**Units**	**IISG group n = 46**	**IIDSG group n = 29**
Operation time	Hours	3.8 (0.9)	5.8 (0.8)
Hospital stay	Days	4.2 (0.8)	5.4 (1.2)
Duration of follow up	Months	30.2 (9.6)	12.7 (5.3)
Intraoperative complications	Number	Nil	4*
Postoperative complications	Number	Nausea - 12	Nausea – 6
Long term complications	Number	Nil	B_12_ deficiency – 3

Data given as mean (S.D) * Explained in the results section.

**Table 4:** **Comparison between the groups according to their body mass index**

		**IISG group (N = 46)**		**IIDSG group (N = 29)**	
		**Obese**	**Non Obese**	**Obese**	**Non Obese**
Number of Patients	No	32	14	21	8
Remission of DM	No (%)	17 (53)	4 (47)	16 (55)	5 (45)
Baseline BMI	kg/m^2^	31.2(4.7)	24.7(5.3)	33.5(2.6)	25.3(1.4)
BMI at end point	kg/m^2^	27.1(4.8)	23.5 (6.4)	27.5(4.6)	22.9 (5.1)
Δ Body weight	kg	9.7 (6.6)	1.4(2.3)	7.8(5.4)	2.2 (1.7)
DRS	Score	8.1 (0.8)	10.2 (0.9)	9.7 (0.8)	12 (0.5)

*BMI* Body Mass Index, *Δ Body weight* Change in the weight from baseline, *DRS* Diabetes Remission Score.

Mean DRS in patients who underwent IIDSG (n = 29) was 10.4 ± 1.3 (Range: 8–14, significantly higher than II + SG group, p = 0.0004). Twenty one (72%) had a remission in diabetes. DRS was significantly lower in patients with remission than patients without remission (9.7 ± 0.8 versus 12.0 ± 0.5, p < 0.0001). All the patients with DRS 8–10 achieved remission, while 55% patients with DRS 11 had diabetes remission. There was no remission in diabetes in patients with baseline DRS ≥ 12.

Patients with DRS ≥ 10 in IISG group and DRS ≥ 12 in IIDSG group did not get into remission. DRS was not significantly different (p = 0.1468) in patients without remission in IISG (10.2 ± 0.9) versus patients with remission in IIDSG (9.7 ± 0.8). In patients with IIDSG, intraoperative complications were observed in 4 patients. One patient had a dusky duodenal stump leading to application of the drain. Another patient had a suture-passer tip breakage into the abdominal wall which was extricated by C arm. A 2 cm opening at the gastric antrum in another patient was closed in 3–0 PDS because of faulty stapling and the last patient had ileal perforation managed by exploratory laparotomy and closure. At 3 months postoperative follow up, none of these patients had any complications with regard to the intraoperative and immediate postoperative events.

## Discussion

Our study focuses on the development of a novel score (DRS) preoperatively in predicting diabetes remission following IISG or IIDSG. DRS was derived from the variable factors predicting the remission in diabetes. Our previous studies on IISG have demonstrated duration of diabetes, BMI and stimulated C-peptide response as baseline parameters of response [14,15]. Previous studies of RYGB reported that old age, longer duration of diabetes, lower C-peptide level, and lower BMI reduce the likelihood of response to surgery [7,20,21]. In type 2 diabetes patients with duration of diabetes < 5 years, a resolution of the disease was obtained in 95%, whereas the rate of resolution was only 75% and 54% in those who had type 2 diabetes for 6–10 or >10 years [5]. Scopinaro et al. has shown that metabolic surgery is less effective in individuals with lower BMI [22,23]. Insulin resistance is a major contributing factor for diabetes in patients with high BMI, whereas beta cell dysfunction predominates in the normal weight individuals. Hence, the benefits of metabolic surgery are observed more in obese individuals when compared to nonobese patients. Our data also showed similar results in both the groups as shown in Table 4. IIDSG gives augmented stimulus to the enteroinsular hormonal axis leading to better resolution of diabetes. The baseline body weight and the amount of excess body weight lost determine the benefit rates in diabetes resolution. Previous reports suggest that diabetes remission is seen in 60% of patients with restrictive procedures and up to 80 – 90% in combined (restrictive and malabsorptive) procedures [6,23]. Advanced age is an important consideration for patient selection also, because of the augmented risks with surgery and weight loss [24,25].

DRS was significantly lower in patients with remission than patients without remission. This demonstrates that patients with lower baseline DRS have better remission rates and patients with DRS of 7–8 should only be subjected to IISG. Our data demonstrate that IIDSG may be taken up for patients with a DRS up to 11. Additionally DRS was not significantly different (p = 0.1468) in patients without remission in IISG (10.2 ± 0.9) versus patients with remission in IIDSG (9.7 ± 0.8). This indirectly suggests that IIDSG instead of IISG would have helped them in achieving remission.

IIDSG is more beneficial over the conventional IISG procedure due to the following points. The IIDSG excludes the duodenum, leading to attenuated release of Rubino’s factor (anti-incretin factor, which promotes insulin resistance) and Gastric Inhibitory Polypeptide (GIP) [26]. Disruption or attenuation of GIP action is associated with decreased glucagon release, diminished weight gain, resistance to diet-induced obesity, and improved insulin sensitivity in preclinical studies [27-30], whereas genetic variation within the human Gastric Inhibitory Polypeptide Receptor (GIPR) gene is linked to control of postprandial glucose and body weight [31,32]. Exclusion of duodenum also leads to abolition of hedonic (pleasure) value of food, resulting in lesser indulgence in feeding [33]. In comparison to IISG, the interposed ileal segment is shifted more proximally in IIDSG, leading to augmented glucagon like peptide (GLP-1) hormone secretion [16,17]. Our surgical technique has no risk of malabsorptive complications as the procedure does not involve removal of either intestinal digestive or absorptive surface. The previous published long term follow up studies using this technique also did not report any malabsorptive complications. Iron malabsorption is theoretically possible due to lack of gastric intrinsic factor after the surgery. However, we prescribed supplemental iron to all patients postoperatively preventing this complication.

The surgical technique used in this study is designed essentially for diabetes control and utilizes both the foregut and hindgut mechanisms [34]. The SG component restricts calorie intake and reduces ghrelin [35]; it also speeds up gastric transit of food, reaching the ileal segment faster. The GLP-1 response is defective in T2DM leading to diminished first phase of insulin secretion [36]. The ileal interposition helps in the rapid stimulation of ileal segment by ingested food leading to augmented GLP-1 secretion [13-15]. GLP-1 also influences glucose metabolism by inhibiting glucagon secretion, decreasing hepatic gluconeogenesis, delaying gastric emptying, promoting satiety, suppressing appetite, and stimulating glycogenesis [37-43].

We propose a baseline assessment of DRS as a possible predictor of remission in diabetes following IISG or IIDSG. The DRS may be divided into 3 grades as grade 1 (mild, DRS 7–8), grade 2 (moderate, 9–11) and grade 3 (severe, DRS 12–14). Higher scores indicate a diminished chance of achieving remission in a given patient. The advantages of DRS include an easy clinically relevant score, combining multiple factors affecting the diabetes and give an objective measure for comparison between the procedures. Our study has certain limitations. The study analysis and the development of DRS were based on retrospective observational analysis of the data. The DRS score was developed based on the data from sleeve gastrectomy and ileal interposition. Hence, DRS may not be useful for predicting remission of other bariatric or metabolic procedures. The status of obesity alone as a determinant of metabolic surgery is incorrect due to the identification of a phenotype called metabolically obese and normal weight individuals [44]. This also limits the wide application of this scoring system prior to the surgery. The study was inadequately powered to give a definite conclusion due to limited number of the patients and mixed population in each group. Moreover, our data from a single center may not represent the entire diabetes population of India.

## Conclusion

Diabetes remission score is a useful tool for predicting the remission in patients with type 2 diabetes undergoing IISG or IIDSG. We propose to introduce DRS as a tool to select the type of surgical procedure and to predict the postoperative diabetes remission. Further multicenter studies of patients from various ethnicities are required to confirm our preliminary observations.
